# Observation Variation in Ultrasonography Assessment of Thyroid Nodules

**DOI:** 10.22038/AOJNMB.2021.59283.1411

**Published:** 2022

**Authors:** Yasaman Sharifi, Susan Shafiei, Hamed Tabesh, Behzad Aminzadeh, Parvaneh Layegh, Abolfazl Mahmoodzadeh, Seyed Rasoul Zakavi, Saeid Eslami

**Affiliations:** 1 Department of Medical Informatics, Faculty of Medicine, Mashhad University of Medical Sciences, Mashhad, Iran; 2 Nuclear Medicine Research Center, Mashhad University of Medical Sciences, Mashhad, Iran; 3 Department of Radiology, Faculty of Medicine, Mashhad University of Medical Sciences, Mashhad, Iran; 4 Department of Radiology, Faculty of Medicine, Neyshabur University of Medical Sciences, Neyshabur, Iran; 5 Pharmaceutical Research Center, Mashhad University of Medical Sciences, Mashhad, Iran; 6 Department of Medical Informatics, Amsterdam UMC (location AMC), University of Amsterdam, Amsterdam, The Netherlands

**Keywords:** Inter-observer agreement Intra-observer agreement Observation variation Ultrasonography, Thyroid nodules

## Abstract

**Objective(s):**

Accurate detection and competent management of thyroid nodules, as a common disease, basically depends on the reliability of the ultrasonography (US) report. In this research, we evaluated inter and intra-observer variation among ultrasonography reporters, based on ACR-TIRADS.

**Methods:**

In this retrospective study, 345 thyroid US images of 150 patients were reviewed. Three clinicians with at least 6-year experience in thyroid US reviewed the images twice at 6-8 weeks’ intervals. Composition, echogenicity, shape, margin, and echogenic foci based on ACR-TIRADS were reported, independently. Inter and intra-observer variations were calculated based on Cohen’s Kappa statistics.

**Results:**

345 ultrasonography images of 150 patients with thyroid nodules (83 women and 67 men) with a mean age of 65 years were reviewed. Moderate to the substantial intra-observer agreement was achieved with the highest Kapa value in the category of shape (k=0.61-0.77). For TIRADS level, the moderate intra-observer agreement was observed (k=0.42-0.46). Inter-observer agreement for the US category of thyroid nodules was obtained slightly to moderate. Composition (k=0.42 and 0.51) and echogenicity (k=0.45 and 0.46) showed the highest overall agreement and margin showed the lowest overall agreement (k=0.18 and 0.19). In assessing TIRADS level of nodules, a fair agreement was obtained (k=0.23 and 0.29).

**Conclusion:**

Moderate to substantial intra-observer agreement and slight to moderate inter-observer variation for evaluation of thyroid nodules; shows the need for a computer-aided diagnosis system based on artificial intelligence to assist our physicians in differentiating thyroid nodule characteristics based on explicit image features. An additional training course based on ACR-TIRADS for physicians can be another useful recommendation.

## Introduction

 Thyroid nodules are a common medical problem with higher frequency in women and the elderly (1). The prevalence of palpable thyroid nodules in the general population is reported at 5% in women and 1% in men and is increasing around the world (2). However, high- resolution ultrasonography evaluation in randomly selected healthy individuals showed an incidence of 16-98% for incidentalomas (1-3). Since 10-15% of thyroid nodules are reported to contain cancerous cells, accurate evaluation of thyroid nodules and defining the possibility of cancer is a principal part of thyroid cancer management (2-5). 

 Ultrasonography (US) is an available and cost-effective method for thyroid nodules detection and evaluation. Not only can the US detect small nodules, but also it can be useful in differentiation between benign and malignant nodules. Furthermore, it is helpful as a guide for fine-needle aspiration (FNA) of non-palpable or posteriorly located lesions (6, 7). On the other hand, thyroid US as a highly subjective and operator-dependent method may lead to significant variations in reporting, which can affect the management of nodules (8, 9).

 For better evaluation of thyroid nodules and making standard reports, several researchers have recommended the Thyroid Imaging Reporting and Data System (TIRADS) as a standard risk stratification protocol (10). Five of these systems have been approved by international scientific institutions and among them, the American College of Radiology (ACR) has introduced ACR-TIRADS that indicated the best performance (11). ACR-TIRADS is a worldwide acceptable system for reducing inter and intra-observer variability. This standard system considers 5 categories including composition, echogenicity, shape, margin, and echogenic foci.

 Despite these efforts, some authors have reported low levels of reproducibility in thyroid US reports (8, 10). There are many researches about similarities and differences between two interpretations of one thyroid nodule, but the reproducibility has remained under assessment. In this retrospective study, we evaluated inter and intra-observer variability of US evaluation in thyroid nodules based on ACR-TIRADS.

## Methods

***Study population***

 We retrospectively selected 345 ultrasono-graphy images of thyroid nodules in 150 patients (up to three nodules in each patient) who were referred for US evaluation of thyroid nodules. The images had been acquired from a Cohort study in 2019-2020 and saved in our thyroid data center for follow-up evaluation and we randomly selected saved images based on our inclusion criteria. The inclusion criteria for select images were 1: images without Color Doppler Flow, 2: Images with at least one thyroid nodule. Patients with history of thyroid surgery or sub-acute thyroiditis were excluded. There were 83 (55.3%) women and 67 (44.7 %) men in these patients. The mean age of the patients was 56 years old (range, 31–81 years). The mean size of the nodules was 1.36 mm (range, 0.23-8.90 mm).

***Thyroid US Examination and Retrospective Review***

 To minimize the effect of machines on image interpretation, all examinations were performed and saved by a Philips affinity 50G Ultrasound Machine (12.5 MHz linear transducer 5 cm).

 We developed a web application for reporting and scoring the images based on ACR-TIRADS independently by three physicians (two radiologists and one nuclear medicine specialist) with 10, 9, and 6 years of experience in thyroid imaging and none of these clinicians were in the process of collecting or observing the images”.

One view of a thyroid image in a transverse or longitudinal plan was used for assessment. The readers were blinded to the patients’ history and demographic characteristics.

**Figure 1:**
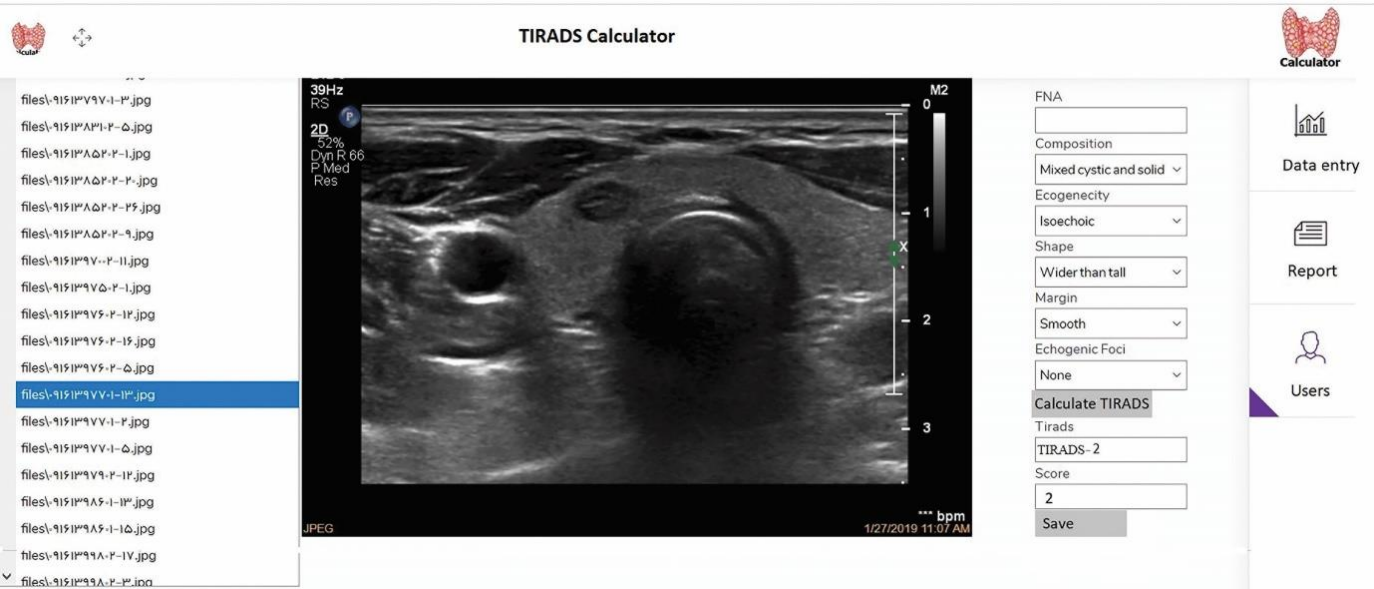
A screenshot of the TIRADS calculator system

 This application was implemented using the C# language in the ASP.NET technology and SQL Server database. The application was available online to three physicians via a user panel to assessing and scoring the images in the simplest and fastest way possible. In addition to the user panel of physicians, this system also has a management panel for the site administrator, which has features such as managing users and receiving reports. A screenshot of our system is shown in Fig1. The menu on the left side of the screen includes an image list and selected image shows in the center of the screen and the right-hand section contains the ACR-TIRADS category that physicians can simply select the appropriate item of related category from Combo Box and then click the “Calculate TIRADS” button to calculate the score and TIRADS level automatically and then click “Save” button to save the information to the database.

 The criteria which should be determined by all reporters identified by the ACR-TIRADS scoring system including composition, echogenicity, margin, shape, and echogenic foci.

 Composition refers to the Internal content of a nodule, which is classified based on the ratio of the cystic and solid part of the nodule. Echogenicity has described the brightness of a thyroid nodule in comparison with surrounding thyroid tissue. The margin was referred to as the boundary between the nodule and the adjacent thyroid parenchyma. The shape was described as the ratio of posterior-anterior diameter to the horizontal diameter of nodules in the transverse plane. Echogenic foci were referred to the central region in the nodule that has very high echogenicity in comparison to the surrounding thyroid tissue (8). Each ACR-TIRADS category and TIRADS level/ Management based on the earned score is shown in Table 1 and Table 2 respectively.

 To evaluate intra-observer variation, all three physicians reviewed the slides again after 6 weeks, using the same method. No explanation of the descriptors, information about the previous reports, or new education was given. They were asked to make the second report with the same system and the same criteria.

**Table 1:** ACR-TIRADS category and sub-category and their score

**ACR-TIRADS Category**	**Sub-category (score)**					
**Composition**	Cystic or almost completely cystic (0)	Spongiform (0)	Mixed cystic and solid (1)	Solid or almost completely solid (2)		
**Echogenicity**	Anechoic (0)	Hyperechoic (1)	Isoechoic (1)	Hypoechoic (2)		Very hypo echoic (3)
**Shape**	Wider than tall (0)	Taller than wide (3)				
**Margin**	Smooth (0)	Ill-defined (0)	Lobulated (2)	Irregular (2)	Extrathyroidal extension (3)	
**Echogenic Foci**	None or Large comet-tail artifacts (0)	Macrocalcifications (1)	Peripheral calcifications (2)	Punctate echogenic foci (3)		

**Table 2:** TIRADS level and Management of nodule based on a nodule’s ACR TIRADS level and its maximum diameter

	**score**				
	0	2	3	**4-6**	**7 or more**
**TIRADS Level**	TR1 (Benign)	TR2 (Not Suspicious)	TR3 (Mildly Suspicious)	TR4 (Moderately Suspicious)	TR5 (Highly Suspicious)
**Management**	No FNA	No FNA	FNA if>=2.5cm Follow if >=1.5cm	FNA if>=1.5cm Follow if >=1cm	FNA if>=1cm Follow if >=0.5 cm

***Statistical analysis***

 All data were recorded and analyzed using SPSS software, version 26.0. Descriptive analysis was done using frequency tables, mean and standard deviation.

 For assessing Intra and inter-observer agreement of each ACR-TIRADS category of nodules between the 3 observers, Cohen kappa and Fleiss’ Multi-rater Kappa statistics were used respectively. Landis and Koch suggested the relationship between the kappa values and the level of agreement (Table 3) (12).

 For all statistics, 95% confidence intervals (CI) were also calculated. P-values less than 0.05 were considered statistically significant.

**Table 3:** Relationship between the kappa values and the level of agreement based on Landis and Koch suggestion (12)

**kappa values**	**level of agreement**
0 – 0.20	slight agreement
0.21 – 0.40	fair agreement
0.41 – 0.60	moderate agreement
0.61 – 0.80	substantial agreement
0.81–1.00	perfect agreement

## Results

 Intra-observer agreement among the three reviewers for each US category based on ACR-TIRADS classification was summarized in Table 4.

 Overall, moderate to the substantial intra-observer agreement was achieved.

The highest kappa value and substantial intra-observer agreement were obtained for the evaluation of the shape of the nodule (k=0.61-0.77).

 Composition and echogenicity of the nodule achieved moderate to the substantial intra-observer agreement (k=0.59-0.66 and k=0.52-0.65 respectively). Composition and echogenicity of the nodule achieved moderate to the substantial intra-observer agreement (k=0.59-0.66 and k=0.52-0.65 respectively). Which, of course, these agreements were at the lower range of substantial and closer to the moderate agreement.

 Moderate intra-observer agreement was noted in assessing Margin, (k=0.41-0.45) and echogenic foci in the lesion (k = 0.49-0.59). 

Table 4 shows that moderate intra-observer agreement was found for TIRADS categori-zation (k=0.42-0.46).

 Inter-observer agreement for each US characteristic based on ACR-TIRADS classification is summarized in Table 5.

**Table 4:** Intra-observer variation in thyroid nodule assessment based on ACR-TIRADS with Cohen kappa measurement

Cohen kappa*	**Observer 1**	**Observer 2**	**Observer 3**
**ACR-TIRADS ** **Categories**			
**Composition**	0.66(0.03,0.0001)	0.60 (0.04, 0.0001)	0.59 (0.04, 0.0001)
**Echogenicity**	0.65(0.03,0.0001)	0.61 (0.04, 0.0001)	0.52 (0.04, 0.0001)
**Shape**	0.77(0.09,0.0001)	0.61(0.06,0.001)	0.62 (0.14,0.0001)
**Margin**	0.45(0.08, 0.0001)	0.41(0.04,0.0001)	0.44(0.04,0.0001)
**Echogenic Foci**	0.57(0.06, 0.0001)	0.59(0.04, 0.0001)	0.49(0.04, 0.0001)
**TIRADS level**	0.46(0.03,0.0001)	0.42(0.03,0.0001)	0.45(0.03,0.0001)

* Value (Standard Error, P value)

**Table 5:** Inter-observer variation in thyroid nodule assessment based on ACR-TIRADS with Fleiss Multirater Kappa measurement

**ACR-TIRADS Categories**	**Descriptors**	**First FM-Kappa (SE, P value)**	**Second FM-Kappa (SE, P value)**
Composition	Cystic or almost completely cystic	0.50 (0.03, 0.0001)	0.60 (0.03, 0.0001)
	Mixed cystic and solid	0.33 (0.03, 0.0001)	0.44 (0.03, 0.0001)
	Solid or almost completely solid	0.53 (0.03, 0.0001)	0.59 (0.03, 0.0001)
	Spongiform	0.21 (0.03, 0.0001)	0.30 (0.03, 0.0001)
	Overall Agreement	0.42 (0.02, 0.0001)	0.51 (0.02, 0.0001)
Echogenicity	Anechoic	0.50 (0.03, 0.0001)	0.60(0.03, 0.0001)
	Hyperechoic	0.49 (0.03, 0.0001)	0.38(0.03, 0.0001)
	Hypoechoic	0.47 (0.03, 0.0001)	0.49(0.03, 0.0001)
	Very hypoechoic	-0.014 (0.03, 0.658)	-0.015 (0.03, 0.631)
	Isoechoic	0.41 (0.03, 0.0001)	0.40(0.03, 0.0001)
	Overall Agreement	0.45 (0.02, 0.0001)	0.46(0.02, 0.0001)
Shape	Wider-than-tall	0.34(0.03,0.0001)	0.10(0.03,0.0001)
	Taller-than-wide	0.34(0.03,0.0001)	0.10 (0.03,0.0001)
	Overall Agreement	0.34(0.03,0.0001)	0.10 (0.03,0.0001)
Margin	Smooth	0.19(0.03,0.0001)	0.20(0.03,0.0001)
	Lobulated	0.18(0.03, ,0.0001)	0.44(0.03,0.0001)
	Extrathyroidal extension	0.08(0.03,0.0001)	-0.009(0.03, 0.775)
	Ill-defined	0.20(0.03,0.0001)	0.17(0.03,0.0001)
	Irregular	0.03(0.03, 0.0001)	-0.012(0.03, 0.702)
	Overall Agreement	0.18(0.02, 0.0001)	0.19(0.02, 0.0001)
Echogenic Foci	None	0.45(0.03, 0.0001)	0.46(0.03, 0.0001)
	Macrocalcification	0.31(0.03, 0.0001)	0.22(0.03, 0.0001)
	Peripheral (rim) calcifications	0.69(0.03, 0.0001)	0.66(0.03, 0.0001)
	Punctate echogenic foci	0.32(0.03, 0.0001)	0.18(0.03, 0.0001)
	Large Comet-tail artifacts	0.16(0.03, 0.0001)	0.16(0.03, 0.0001)
	Overall Agreement	0.38(0.02, 0.0001)	0.32(0.02, 0.0001)
TIRADS level	TIRADS-1	0.23(0.03, 0.0001)	0.43(0.03, 0.0001)
	TIRADS-2	0.20(0.03, 0.0001)	0.30 (0.03, 0.0001)
	TIRADS-3	0.33(0.03, 0.0001)	0.33(0.03, 0.0001)
	TIRADS-4	0.17(0.03, 0.0001)	0.21(0.03, 0.0001)
	TIRADS-5	0.21(0.03, 0.0001)	0.20(0.03, 0.0001)
	Overall Agreement	0.23(0.02,0.0001)	0.29(0.02,0.0001)

 We achieved slight to moderate inter-observer agreement for the US category of thyroid nodules. Composition and echogenicity showed the highest overall agreement and margin showed the lowest overall agreement. Composition (k=0.42 and 0.51) and echogenicity (k=0.45 and 0.46) achieved moderate inter-observer agreement and margin obtained slight agreement (k=0.18 and 0.19).

 In assessing composition, ‘Spongiform’ and ‘Mixed cystic and solid’ characteristics had the lowest kappa value and ‘Cystic or almost completely cystic’ had the highest inter-observer agreement. Categorization in ‘Spongiform’ had a fair inter-observer agreement (k=0.21 and 0.30) while ‘Mixed cystic and solid’ categorization showed a fair to moderate agreement (k=0.33 and 0.44) and ‘Cystic or almost completely cystic’ characteristics showed moderate agreement (k=0.50 and 0.60).

 In the evaluation of echogenicity, there was more inter-observer agreement for categorization in the ’Anechoic’ group than in other groups of echogenicity (k=0.50 and 0.60) and inter-observer agreement for ‘Very hypoechoic’ was not significant because the number of samples in this group was very small.

For the “shape” of nodules, in the first assessment, fair inter-observer agreement was seen (k=0.34) while in the second assessment slight agreement was obtained (k=0.11).

 For “margin” evaluation, and overall inter-observer agreement was slight (k=0.18 and 0.19). In the second assessing agreement for categorization in ‘Extrathyroidal extension’ and ‘Irregular’, there was not any inter-observer

agreement.

 In assessing Echogenic Foci, overall inter-observer agreement was fair (k=0.38 and 0.32), in this category ‘Peripheral calcifications’ showed substantial agreement and the highest kappa value (k=0.69 and 0.66) and ‘Large Comet-tail artifacts’ showed the slight agreement and the lowest kappa value (k=0.16 and 0.16).

 In assessing TIRADS level of nodules, the fair intra-observer agreement was obtained (k=0.23 and 0.29).

**Figure 2:**
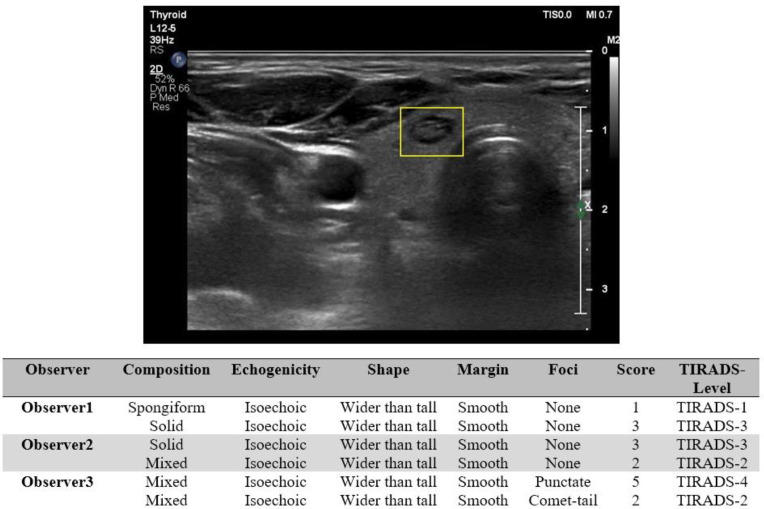
First example of the nodule with the low agreement in some features

**Figure 3:**
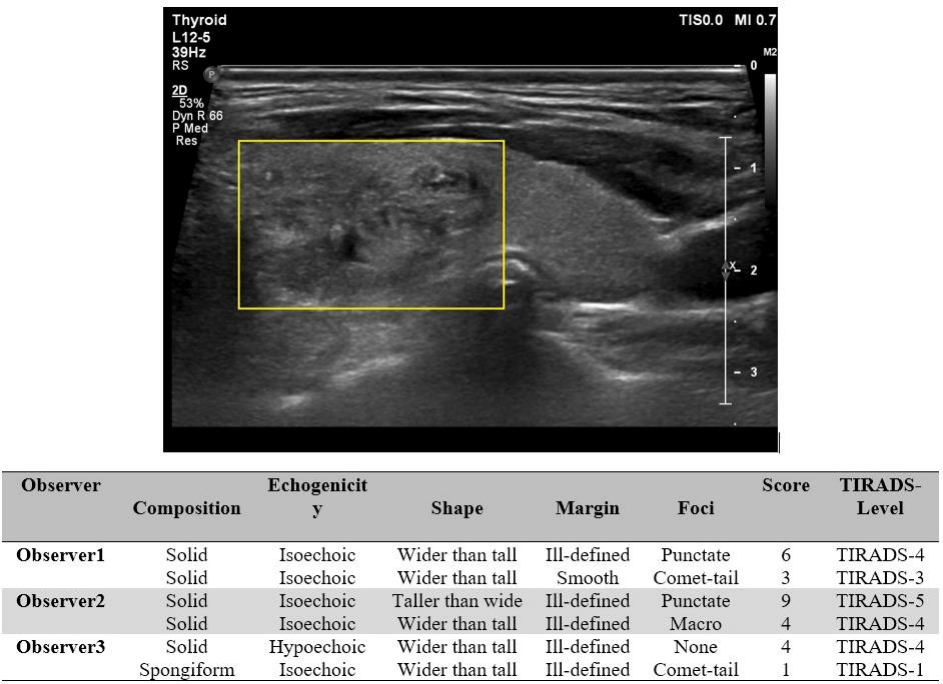
Second example of the nodule with the low agreement in some features

Figures 2 and 3 represent examples of the nodule with low agreement in some features.

## Discussion

 There have been many researches on observer variability of thyroid nodule volumetric and phantom studies. However, inter-observer agreement on thyroid nodules was assessed in just a few studies. Choi et al performed their research on 204 thyroid nodules and assessed intra and inter-observer agreement between 4 radiologists with more than 5-year experience (6). The great point of this study backs to the preparation of 2-4 grey-scale and color Doppler images and at least one transverse and one longitudinal image of each nodule, which can result in better interpretation and higher agreement. They claim that the intra-observer agreement was almost all substantial (Kapa value>0.61). For inter-observer variations, they achieve fair to a substantial agreement with the highest value in shape and vascularity (K=0.61 and 0.64, respectively). Our study showed moderate to the substantial agreement in intra-observer variability. Although we selected well-experienced physicians with at least 6 years of experience in thyroid imaging and asked them for reporting based on ACR TIRADS, the overall agreement was not perfect even in intra-observer evaluation. Substantial intra-observer agreement was obtained for composition, echogenicity, and shape. However, among more important criteria including, margin, echogenic foci, and TIRADS level, all reviewers showed moderate intra-observer agreement. These 

findings suggest that margin and echogenic foci are not well-agreed and well-trained criteria for our observers. The other explanation for these results can be due to the method of the study. We provided just one image of every nodule and some criteria might be difficult to be evaluated with one image only.

 In assessing nodular margin, moderate agreement (k=0.41-0.45) was obtained when categorized into five groups. Considering the ACR-TIRADS system, smooth and ill-defined categories have been scored as zero. Also, lobulated and irregular margins are categorized as score 2. Therefore, in the second step, we classified the margin into three groups based on ACR-TIRADS (scores o, 2, and 3) and analyzed the new data. Our result showed no increase in Kapa value and we confirmed that intra-observer agreement on margin category is not more than moderate level.

 Intra-observer agreement on TIRADS level, the most important category for final classification and introducing a nodule as benign, not suspicious, mildly suspicious, moderately, and highly suspicious was achieved moderate agreement (k=0.42-0.46).

 In another study, Grant et. al showed moderate to the substantial agreement between two independent reporters (K=0.47-0.61). They assessed thyroid nodules based on different systemic categorizations and have resulted in better agreement than us (10). In our study, the inter-observer agreement was slight to moderate in assessing thyroid nodules, as a whole. Again, composition and echogenicity showed the highest Kapa value which confirms that our observers are more familiar with these characteristics. In composition criteria, the spongiform category showed the lowest agreement among the three observers. This may be due to the fact that no exact description has been introduced for this feature and it can be mistaken with the category of ‘Mixed cystic and solid’ when the observer has to decide on just one image. Among different categories of echogenicity, the anechoic pattern showed the highest value which is more likely due to the clear feature of this category. A very hypoechoic pattern showed the fewest number and the lowest Kapa value. We believe that this category is not usually considered in daily reports and may have been categorized in the hypoechoic group by the reviewers. Other categories of composition and echogenicity showed moderate inter-observer agreement.

 The category of shape showed fair and slight Intra-observer agreement in the first and second observations. This low intra-observer agreement can be due to the fact that no measurement tool was available for our reporters and they had to subjectively define two dimensions of the nodule. If real images and size instruments were accessible for our physicians, higher Kapa values would be expected.

 Shape and margin showed slight to a fair agreement with lower Kapa values for more uncommon patterns like irregular border and extra thyroid extension. As we looked through general thyroid US reports in our country, despite composition and echogenicity, these categories are not usually reported by our physicians. Therefore, we concluded that although our physicians have been learned all ACR-TIRADS categories in the educational courses they may not have got the expertise for less common characteristics that are not seen in their daily practice.

 In the category of echogenic foci, the overall inter-observer agreement was fair (k=0.38 and 0.32). In this category ‘Peripheral calcifications’ showed substantial agreement and the highest kappa value (k=0.69 and 0.66) and ‘Large Comet-tail artifacts’ showed the slight agreement with the lowest kappa value (k=0.16 and 0.16).

 The highest agreement in the Peripheral calcification pattern showed that our observers are relatively familiar with this category. On the other hand, they do not have any threshold size 

for macro and microcalcification and any measurement tool for exact size determining of the calcification foci. So, some borderline calcification foci may locate in either macro or microcalcification categories. The lowest Kapa value was obtained for the comet-tail artifact. We reviewed all data and concluded that since this artifact cannot change the TIRADS score, our observers did not consider it as important.

 As the last step, we calculated the Kapa value for the TIRADS level and observed that fair agreement was achieved for this important factor. As we know, the TIRADS level, as an effective value in clinical decisions, is the most important part of thyroid US reports. Clinicians try to make the best decision for their patients based on the TIRADS level. With these variable interpretations of thyroid nodules with different or inaccurate TIRADS scores, which is the major limitation of ultrasonography, decision-making may lead to under or over treatment of the patients. In patients with under-diagnosis of thyroid nodules, numerous difficulties in follow-up and surgical complications may be increased. On the other hand, over staging of thyroid nodules lead to higher rates of unnecessary FNA and over-care problems such as considerable anxiety in patients, which in turn result in a significant burden on the health care system.

 In general, our three physicians had been educated in three different institutes and reported based on their experience and low levels of agreement can be expected.

 The essential recommendation to prevent such observer variations is the use of computer-aided diagnosis (CAD) systems that provide physicians a second opinion for more accurate characterization of the nodules and differentiate between malignant and benign nodules or classify them into 5 categories of ACR-TIRADS based on explicit image features. Thyroid computer-aided diagnosis (CAD) based on artificial intelligence may further improve diagnostic reliability and reduce intra and inter-observer variability (13, 14). Another recommendation to decrease the effect of individual experiences is an additional training course based on ACR-TIRADS for physicians who are supposed to perform and report thyroid US by one educational system.

 There were some limitations in our study. First, we selected three observers with different educational systems. Second, this study was performed using just one image for each nodule. Our results may have shown higher agreement if we had included more images of one nodule.

## Conclusion

 Moderate to substantial intra-observer agreement among three physicians with more than 6-year experience and slight to moderate inter-observer agreement on thyroid ultrasono-graphy, indicates the essential need for a computer-aided diagnosis (CAD) system to assist our physicians in diagnosis and risk level stratification of ultrasound thyroid nodules. A CAD system not only could decrease intra and inter-observer variations, but also it may help clinicians to make the best decision on the management of thyroid nodules. Another recommendation is additional training course based on ACR-TIRADS by one educational system with a uniform approach to the US description of thyroid nodules to decrease the observational variations.
